# Happiness, Not Awe, Makes People More Diversity‐Oriented

**DOI:** 10.1002/ijop.70270

**Published:** 2026-09-18

**Authors:** Jens Agerström, Magnus Carlsson, Towe Nilsson

**Affiliations:** ^1^ Department of Psychology Linnaeus University Kalmar Sweden; ^2^ Department of Economics and Statistics Linnaeus University Kalmar Sweden; ^3^ Department of Behavioural Sciences and Learning Linköping University Linköping Sweden

**Keywords:** awe, diversity attitudes, emotion induction, happiness

## Abstract

The current research aimed to examine whether experiencing awe has the potential to make people more positively inclined towards diversity. Awe was contrasted with effects of happiness, another positive and potentially perspective broadening emotion. Specifically, we examined the effect of experimentally induced awe on diversity attitudes, diversity fatigue, and an individual difference variable negatively associated with diversity: social dominance orientation (SDO) and compared this to the effects of a happiness induction. In a large‐scale between‐subjects experiment (*N* = 3196), participants were randomly assigned to view an awe‐inducing, happiness‐inducing, or neutral film clip. We found that happiness consistently enhances diversity attitudes, reduces fatigue, and lowers SDO, while awe did not show any effects on the outcomes. The findings suggest that happiness, rather than awe, may be a more effective emotional lever for promoting diversity and inclusion.

## 1 Introduction

Natural wonders, panoramic views, sublime art, and marvellous feats of engineering are some of the extraordinary things that may elicit the emotion of awe in humans (Keltner and Haidt 2003; Shiota et al. 2007). Awe is typically elicited by perceptual vastness that transcends the observer's usual frame of reference in some dimension or domain (Keltner and Haidt 2003; Shiota et al. 2007). It decreases attention to the individual self and enhances people's perception of the world as interconnected and meaningful, broadens perspectives and horizons, and leads to a stronger willingness to engage with other people (Piff et al. 2015). Given these self‐transcending and self‐diminishing features of awe, the question we ask in the current research is whether awe also makes people more positively inclined towards social diversity, equality among groups, and group tolerance. We furthermore compare the potential effects of awe with another positive, perspective broadening emotion—happiness. Feelings of happiness have been shown to broaden people's perspectives, promote concern for others, and encourage helping and cooperative behaviours (van Kleef and Lelieveld 2022). Thus, there are reasons to think happiness could be a potent comparison to the self‐transcendent and self‐diminishing aspects of awe.

While diversity, equity, and inclusion (DEI) initiatives have become rather common in organizations (e.g., diversity training) and in broader society (e.g., the Black Lives Matter movement), resistance to such efforts is also a real and significant phenomenon that seems to have increased as a result of recent changes in the political landscape of some Western countries. To illustrate, the Trump administration recently rolled back DEI programs and initiatives through executive orders. Changes associated with diversity have psychological components which may challenge or even threaten convictions, values, social identities, or ways of interacting with others (Wiggins‐Romesburg and Githens 2018). Resistance to diversity involves silence, passivity towards discrimination and harassment, hostility, and even violence in the workplace. There are many factors contributing to diversity resistance, such as social identity concerns (‘us versus them’), whereby an individual's own group is perceived as superior to the outgroup (Wiggins‐Romesburg and Githens 2018). Categorical thinking in the form of social stereotypes also contributes (Wiggins‐Romesburg and Githens 2018), whereby out‐groups (e.g., ethnic) are often associated with negative traits that may impede an individual's willingness to both work and socialise with individuals from these groups. Hence, successful interventions aimed at overcoming diversity resistance may need to target ‘us versus them’ categorizations, and associated emotions (e.g., fear, anger) and motivations (e.g., ‘psychological threats’). Indeed, awe has been shown to increase people's identification with broader, more universal categories, such as one human race (Shiota et al. 2007). We theorise that this perceived ‘oneness’ should reduce social identity concerns, ‘us versus them’ thinking, and associated prejudice—factors that may undermine positive attitudes towards diversity. Thus far empirical research on awe has shown that it has various downstream consequences, including an increased sense of social belonging (Piff et al. 2015) and numerous prosocial behaviours, such as increased generosity (Piff et al. 2015). Essentially, awe seems capable of transforming people by reorienting their lives, goals and values (Keltner and Haidt 2003).

Similarly to awe, happiness tends to be described with perspective broadening qualities. According to Fredrickson's broaden‐and‐build theory, positive emotions broaden peoples' momentary thought–action repertoires (Fredrickson 2004). People tend to be more open to their environment, see more, and do more when they are in a positive mood. As with awe, happiness has been related to dispositional openness (Chung et al. 2019) and cognitive flexibility (e.g., Polat et al. 2022), both with potentially positive outcomes for diversity. Openness has for example been related to an openness to appreciate human diversity and universality and cognitive flexibility seems to play a role for cultural intelligence and cultural diversity (Bernardo and Presbitero 2018). Apart from this, feelings of happiness also tend to strengthen social bonds (Sharma et al. 2025), where happy people tend to spend more time with others, engage in more substantive conversations, thereby fostering social connection. This, along with the prosocial qualities of happiness are thus likely to reflect a more positive orientation towards others, regardless of potential group belongings.

Given the relative stability of personality traits and values, inducing emotions may constitute a viable tool for achieving personal change and growth (Keltner and Haidt 2003). Indeed, a recent correlation study by Zhao et al. (2023) found that trait awe was associated with lower levels of social dominance orientation (SDO; Pratto et al. 1994)—an individual difference variable believed to be a clear diversity antagonist and a reliable predictor of prejudice. Whether there is a causal effect of temporarily induced emotions on diversity, and factors that impede diversity is yet to be determined.

### 1.1 The Current Research

The aim of the current research is to extend prior work on awe by examining whether experimentally induced awe increases positive attitudes towards diversity. In doing so, we also explore whether awe has the potential to counteract diversity fatigue, resistance, and other diversity‐undermining factors. Specifically, we assess the causal effect of awe on diversity attitudes, diversity fatigue, and SDO. We hypothesise (H1) that experimentally induced awe will result in more positive attitudes towards diversity, less diversity fatigue, and lower levels of SDO compared to when no emotion is induced (neutral control condition).

Importantly, the effects of awe will be contrasted with the potential effects of happiness. Although both emotions share similar positive, perspective‐broadening qualities, awe is more self‐transcendent with a diminished focus on oneself—encouraging people to view themselves as part of universal categories with shared commonality. With such qualities, we predict (H2) that awe will lead to more positive attitudes towards diversity, lower diversity fatigue, and reduced levels of SDO compared to happiness. Prior research shows that adults exposed to an awe‐inducing nature video donated more raffle tickets than those exposed to a happiness‐inducing nature video (Piff et al. 2015). Similarly, in children, awe appears to promote prosocial behaviour towards outgroups to a greater extent than happiness (Stamkou et al. 2023). Whether there is an awe superiority effect in the context of diversity is unclear, however, warranting empirical investigation. Because happiness also has perspective‐broadening and prosocial qualities, it should serve as a solid benchmark to which awe could be compared.

In summary, our hypotheses are as follows:

*Awe will result in more positive attitudes towards diversity, less diversity fatigue, and lower levels of SDO compared to when no emotion is induced*.

*Awe will result in more positive attitudes towards diversity, less diversity fatigue, and lower levels of SDO compared to happiness*.

## 2 Methods

In the pre‐registered research plan,^1^ we report how we determined the sample size, data restrictions, manipulations, and measures in the study. It also contains the film clips used as the experimental manipulation and the dataset.

### 2.1 A Priori Power Calculations

A priori power calculations using G*Power indicate that 651 participants per condition would be required to be able to detect a small difference (Cohen's *d* = 0.2, 1 − *β* = 0.95, *α* = 0.05, two‐tailed) between two independent means, amounting to a total sample size of 1953 participants. To have ample margin, we aimed for a much larger sample size (> 3000).

### 2.2 Participants and Design

A between‐subjects experiment was created using Qualtrics software. Participants (*N* = 3215) were recruited from Prolific. A few cases were invalid (e.g., duplicates), resulting in a final sample size of 3196 participants. Participants were randomly assigned to one of three conditions: awe condition (*n* = 1077), happiness condition (*n* = 1064), or neutral/control condition (*n* = 1055). Participants were asked to fill in basic demographic information; the mean age was 40.45 years (SD = 13.52) and 52% were female. To provide a small economic incentive, participants were paid upon completing the study. Participation in the experiment was anonymous. Before taking part in the study, participants provided informed consent, in accordance with the Declaration of Helsinki.

### 2.3 Materials and Procedure

Participants were invited to a study on interpersonal relations. They consented to participate by ticking an informed consent box in Qualtrics. Three different film clips were used to induce awe, happiness, or a neutral emotional state (these were pretested in a pilot study, see below). The film clips contained both pictures and amplifying music and were of relatively short duration (28 s). These started playing after participants had consented to participate in the study. All film clips started with a written welcome message ‘Welcome to this study on interpersonal relations’, followed by a sequence of emotion inducing (or neutral) pictures. The music began playing as soon as the welcome message was displayed. In the awe condition, participants were exposed to pictures depicting e.g., the Apollo 11 moon landing, the universe, vast landscapes, and people showing awe expressions along with majestic music. In the happiness condition, participants were exposed to pictures depicting, e.g., birthday parties, children playing, and people showing expressions of happiness, along with joyful music. In the control condition, participants were exposed to more emotionally neutral pictures e.g., depicting people studying, working, or taking a coffee break, along with neutral background sounds.

Immediately after the experimental manipulation, we measured three aspects pertaining to diversity orientation: diversity attitudes, diversity fatigue/motivation, and SDO. The order of these measurements was randomised; each measurement was calculated as a mean score of its items.

*Diversity attitudes* were measured with the 15‐item Miville‐Guzman Universality‐Diversity Scale, Short Form (M‐GUDS‐S; Fuertes et al. 2000). A universal‐diverse orientation is a concept associated with multicultural awareness and relates to the recognition and acceptance of both similarities and differences among people (Miville et al. 1999). Examples of items include: ‘I would like to join an organization that emphasises getting to know people from different countries’, ‘Getting to know someone of another race is generally an uncomfortable experience for me’ (reverse coded), and ‘Persons with disabilities can teach me things I could not learn elsewhere.’ Ratings were made on a 6‐point scale (1 = strongly disagree; 6 = strongly agree). Cronbach's alpha was 0.86 in the current study (*M* = 4.62, SD = 0.67).

*Diversity fatigue* was measured with the Diversity Fatigue Scale, which captures majority groups' feelings of weariness towards diversity efforts (Smith et al. 2021). Examples of items include: ‘I am tired of hearing about diversity issues on campus’ and ‘I feel annoyed when someone brings up concerns about diversity in academia’. We modified the scale slightly to increase its applicability to broader society by removing the focus on academia. For example, we removed ‘on campus’ and ‘in academia’ from the two items listed above. To this end, we also changed the item ‘I do not want to see any more diversity classes and programs at [my university]’ to ‘I do not want to see any more diversity programs in society.’ Finally, we removed the item ‘I am uneasy that diversity classes are required for students at [my university]’ because it could not easily be modified. Hence, we ended up with five (instead of the original six) items. Ratings were made on a 5‐point scale ranging from ‘not at all true’ to ‘completely true’. Cronbach's alpha was 0.95 in the current study (*M* = 2.05, SD = 1.17).

*SDO* was measured with the original 16‐item SDO scale (Pratto et al. 1994). The SDO is an individual difference measure capturing preferences for inequality, group‐based hierarchies, and discrimination (Pratto et al. 1994). SDO is a clear diversity antagonist and a reliable predictor of prejudice. Examples of items include: ‘Some groups of people are simply inferior to other groups’, ‘To get ahead in life, it is sometimes necessary to step on other groups’, and ‘All groups should be given an equal chance in life’ (reverse coded). Cronbach's alpha was 0.94 in the current study (*M* = 2.44, SD = 1.09).

### 2.4 Pretest of Film Clips

The film clips were pretested on an independent Prolific sample (*N* = 125) to ascertain that they induced the intended emotion. Participants rated how intensely they experienced the following emotions when watching the three film clips: awe, happiness, shame, sadness, guilt, anger, disgust, fear, and surprise. Ratings were made on a 7‐point scale (1 = not at all, 7 = a great amount). All participants rated all three film clips (repeated measures), and the order was counterbalanced.

The results show that the control film clip indeed induced low levels of the measured emotions (column 1 of Table 1), confirming that it is perceived as emotionally neutral. Furthermore, as is evident from the first row, the awe film clip received high average ratings on awe, significantly higher levels of awe than both the control film clip (*p* < 0.001) and the happiness film clip (*p* < 0.001). Similarly, the happiness film clip received high average ratings on happiness (row 2). It induces significantly higher levels of happiness than both the control film clip (*p* < 0.001) and the awe film clip (*p* < 0.001). The results further show that although there is an emotional overlap between the awe and happiness clip, the awe film clip induces significantly more awe than happiness (*p* < 0.001; row 1 and 2 of column 2). Conversely, the happiness film clip induces significantly more happiness than awe (*p* < 0.001; row 1 and 2 of the column 3). This emotional overlap is well aligned with previous research, as awe inductions often include feelings of happiness (e.g., Bai et al. 2017; Piff et al. 2015).

**TABLE 1:** Descriptive statistics showing emotion ratings for the film clips.

Variable	Control clip	Awe clip	Happiness clip
Awe	1.50 (1.13)	4.89 (1.63)	2.55 (1.63)
Happiness	2.30 (1.41)	4.20 (1.52)	5.14 (1.48)
Shame	1.16 (0.63)	1.09 (0.44)	1.11 (0.46)
Sadness	1.42 (0.95)	1.21 (0.68)	1.27 (0.75)
Anger	1.14 (0.47)	1.05 (0.46)	1.12 (0.59)
Guilt	1.22 (0.59)	1.07 (0.32)	1.15 (0.60)
Disgust	1.15 (0.60)	1.09 (0.52)	1.13 (0.60)
Fear	1.22 (0.74)	1.17 (0.44)	1.10 (0.57)
Surprise	1.66 (1.30)	2.84 (1.70)	2.03 (1.37)

*Note: N* = 125. Mean values with standard deviations in parentheses.

In summary, the results from the pilot study confirm that the film clips induce the intended emotions, enhancing the credibility of the experimental manipulations. After the pilot study, the film clips were slightly adapted to fit the current study on interpersonal relations.

## 3 Results

### 3.1 Balance of Experimental Conditions

To demonstrate the internal validity of the experiment, we conduct balance tests to assess whether random assignment to the experimental conditions was successful. Although randomisation is expected to produce equivalent groups on average, imbalances can occur by chance or due to mistakes in the design.

We conduct balance tests using regression models in which the dependent variable is treatment assignment (awe or happiness), and the independent variables are gender, age, and age squared. The results are shown in Table 2. None of the demographic variables significantly predicted assignment to either the awe or happiness condition, and the *R* ^2^ values are near zero in both models. These results suggest that randomisation was successful: participants in the different conditions do not differ systematically in terms of age or gender.

**TABLE 2:** Balance tests of treatment.

	(1)	(2)
	Awe	Happiness
Female	0.012 (0.017)	−0.003 (0.017)
Age	0.003 (0.004)	−0.001 (0.004)
Age2	−0.000 (0.000)	0.000 (0.000)
Constant	0.263*** (0.080)	0.348*** (0.080)
*N*	3196	3196
*R* ^2^	0.000	0.000

*Note:* Each column presents results from a linear probability model where the dependent variable is an indicator for assignment to the awe (column 1) or happiness (column 2) condition. The neutral/control group serves as the reference category. Independent variables include gender (0 = male, 1 = female), age, and age squared. Robust standard errors in parentheses.

*** *p* < 0.01.

** *p* < 0.05.

* *p* < 0.1.

### 3.2 Main Results

Our main results are presented in Table 3. In the first column, we examine whether the emotion induction affects participants' general attitudes towards diversity. Compared to the neutral condition, the awe condition has no significant effect on diversity attitudes. In contrast, the happiness condition has a positive and statistically significant effect (*p* < 0.01), implying that participants exposed to the happiness‐inducing clip show more favourable attitudes towards diversity. On average, their diversity attitude scores were 0.077 points higher on the 6‐point diversity scale. Thus, although the effect size is rather small, the result suggests that happiness can enhance openness for diversity.

**TABLE 3:** Main results.

	(1)	(2)	(3)
	Attitudes towards diversity	Diversity fatigue	SDO
Awe	−0.000 (0.029)	−0.071 (0.051)	−0.062 (0.047)
Happiness	0.077*** (0.029)	−0.100** (0.051)	−0.147*** (0.047)
Constant	4.599*** (0.021)	2.111*** (0.036)	2.399*** (0.033)
*N*	3196	3196	3196
*R* ^2^	0.003	0.001	0.003

*Note:* Each column reports results from OLS regression models where the dependent variable is one of three diversity‐related outcomes: diversity attitudes (column 1), diversity fatigue (column 2), and SDO (column 3). The reference category is the neutral condition. Coefficients for awe and happiness represent differences relative to this group. Robust standard errors in parentheses.

*** *p* < 0.01.

** *p* < 0.05.

* *p* < 0.1.

In the second column, we analyse the impact of the emotion induction on participants' levels of diversity fatigue. Again, compared to the neutral condition, the awe condition does not have an impact, while the effect for happiness is significant (*p* < 0.05), suggesting a modest reduction in fatigue towards diversity in the happiness condition. On average, participants who watched the happiness‐inducing clip scored 0.100 points lower on the 5‐point diversity fatigue scale than those in the neutral condition.

Finally, in the third column, we analyse whether the emotion inductions affected participants' level of SDO—i.e., their preferences for group‐based hierarchies and inequality. Awe had no statistically significant effect compared to the neutral condition. However, in line with the results in column 1 and 2, the happiness condition again shows a significant effect (*p* < 0.01). Relative to the neutral control group, participants who viewed the happiness‐inducing clip scored on average 0.147 points lower on the SDO scale (which runs from 1 to 6).

Overall, the results provided no support for the hypothesis that awe increases diversity orientation. Across all three outcomes—diversity attitudes, diversity fatigue, and SDO—awe did not differ significantly from the neutral control condition. In contrast, happiness showed consistent effects across the outcomes, leading to more favourable diversity attitudes, reduced diversity fatigue, and lower levels of SDO.

### 3.3 Robustness

The dataset contains outliers in participants' response durations, which could be a problem for the validity of the results (e.g., by inflating variance and/or masking or exaggerating true effects). Such outliers may arise from participants who rushed through the study without fully engaging or were distracted during the experiment, etc. To investigate the robustness of our estimates, we repeated the main analysis but excluded participants whose durations fell below the 5th percentile (less than 201 s) or above the 95th percentile (more than 837 s). Excluding outliers did not substantially alter the results (see Table 4). If anything, the pattern of findings observed in the main analyses became slightly stronger.

**TABLE 4:** Robustness.

	(1)	(2)	(3)
	Attitudes towards diversity	Diversity fatigue	SDO
Awe	−0.004 (0.032)	−0.089 (0.055)	−0.079 (0.051)
Happiness	0.080** (0.032)	−0.129** (0.055)	−0.166*** (0.051)
Constant	4.592*** (0.022)	2.149*** (0.039)	2.422*** (0.036)
*N*	2713	2713	2713
*R* ^2^	0.003	0.002	0.004

*Note:* Excluding outliers. This regression mirrors those in Table 2, but participants with response durations below the 5th percentile (< 201 s) or above the 95th percentile (> 837 s) are excluded. See also the notes below Table 3. Robust standard errors in parentheses.

*** *p* < 0.01.

** *p* < 0.05.

* *p* < 0.1.

As shown in the table in Appendix A, the main findings also remain stable when controlling for the demographic variables (gender, age, and age squared).

## 4 Discussion

The current experiment set out to examine whether preference for diversity could be influenced by a momentary induction of awe, more so than induced happiness. The results yielded no support for our hypotheses. In relation to our first hypothesis, participants in the awe condition did not differ significantly from the emotionally neutral control condition regarding attitudes towards diversity, diversity fatigue, or levels of SDO. In relation to the second hypothesis, awe did not make participants more diversity‐oriented than happiness.

Compared to the emotionally neutral condition, the results showed that happiness had a significant effect on all measures, resulting in more positive diversity attitudes, less diversity fatigue, and lower levels of SDO. The positive effect of happiness could be explained by its perspective broadening qualities, which may facilitate the inclusive treatment of members of diverse groups, including people who have historically been excluded or discriminated against. Happiness signals opportunity and can cause a desire to affiliate with other people and to create social bonds (van Kleef and Lelieveld 2022). Research suggests that happiness fosters social bonding, particularly in primary social contexts (e.g., marriage, friendship) but also in more distal contexts (e.g., social participation; Sharma et al. 2025). However, it is still unclear how far these social bonding effects extend, i.e., whether they incorporate more distant social relations, including those with outgroups (Sharma et al. 2025). Additionally, happiness has been found to promote concern for others and increase prosocial behaviours such as helping and cooperation (van Kleef and Lelieveld 2022). Possibly, the prosocial consequence of happiness may explain why happiness increased preference for diversity and inclusion in the current experiment. The reason for this is that prosocial behaviour is positively associated with universalism values which conceptually relate to diversity and inclusion since they involve understanding, appreciation, tolerance, and protection for the welfare of all people (Lönnqvist et al. 2013). Apart from more prosocial tendencies, happiness is also related to lower levels of hostility and anger. This explains the somewhat surprising relationship between brief inductions of happiness and trait measures of SDO, a measure clearly linked to group‐based aggression, hostility and oppression.

Interestingly, there is also correlational research suggesting that diversity seems to be associated with happiness. Countries that report greater happiness tend to be rated as more progressive on dimensions pertaining to diversity, equality, and inclusion (Diener et al. 1995). This relationship also seems to exist within countries. On average, Americans tend to be happier during time periods with less national income inequality compared to time periods with more national income inequality (Oishi et al. 2011). While it seems reasonable to assume that equality and inclusion influence happiness, possibly via mechanisms such as perceptions of fairness and social trust (Oishi et al. 2011), the results from the current study suggest that the reverse causal pattern may also be true, namely that happy people are more likely to be proponents of diversity and inclusion. Possibly, then there is a bidirectional link between happiness and diversity/inclusion.

The link between happiness and diversity is interesting given previous research showing that happiness increases people's reliance on social stereotypes (Bodenhausen et al. 1994), which should counteract diversity. This effect was attributed to happiness induced global processing which ignores individuation information in favour of mental categorisation of people into groups based on traits. Additionally, our results are also contrary to research by Stamkou et al. (2023) which could not show any effect of happiness on outgroup prosociality among children. Possibly, happiness may elicit several different processes, some of which are competing with one another. Although the findings of the current research suggest that diversity facilitating processes win out, future studies may want to identify the relative contribution of happiness induced competing processes (e.g., prosocial values versus stereotyping) for diversity.

Unexpectedly, we also found that happiness resulted in significantly more positive diversity attitudes, and marginally less SDO when compared with awe. This finding is more difficult to explain since it is opposite to what we expected. It does not seem to be explained by differences in the potency of the emotion induction since the pilot study demonstrated that the awe film clip induced similar levels of awe as the happiness film clip induced happiness (means ≈5 on the 7‐point intensity scale).

One possible explanation for this somewhat unexpected finding is the emotional complexity of awe in comparison to happiness. Although often portrayed as a positive emotion, awe can also be based on more negative, threat‐based emotions, which is of importance to diversity measures as threat‐perceptions play a mediating role in the relationship between diversity attitudes and discrimination (Kauff and Wagner 2012). However, our awe film clip induced very small amounts of fear on par with the other film clips. In contrast, it did induce some surprise (*M* = 2.84, SD = 1.70). Although surprise is not necessarily a negatively valanced emotion, it might still affect our results since surprise is an ambiguous emotion that can be related to uncertainty and an increased reactivity to recognise fear (Gordillo León et al. 2021). Furthermore, due to its more challenging, sense‐seeking nature, awe might also generally provoke feelings of anxiety, disorientation and a search for order and coherence (T. Jiang et al. 2024). Such tendencies would likely impede a diversity orientation which draws on curiosity and openness. Thus, although the feelings of surprise in our awe inductions are not necessarily negative, this might still moderate the potential effect of awe on diversity measures.

The happiness clip did not induce such potentially conflicting emotions. Due to its negative relation to both threat perceptions (Horiuchi et al. 2015) and group‐based hostility (Jasielska et al. 2021), happiness might have a less complex relation to diversity orientation. The motivational aspects of happiness might therefore be more easily induced in brief experimental manipulations, whereas more profound inductions may be necessary to reach the more complex, transformative, self‐transcendent motivationalaspects of awe (Jiang et al. 2024).

Another difference between awe and other positive emotions (e.g., joy, pride, amusement) is the feelings of a diminished self that tends to accompany experiences of awe (e.g., Bai et al. 2017; Piff et al. 2015). Such feelings have been found to mediate the relationship between for example awe and increased prosociality (Piff et al. 2015) and collective engagement (Bai et al. 2017). Without a feeling of diminished self, we would therefore not expect to find the same positive effect of awe. As we did not measure this, it is possible that our clip failed to induce such feelings. It is also possible that potential feelings of a diminished self in our study might have been of an overwhelming and isolating nature rather than eye‐opening and expanding. If so, this could reduce the potentially social bonding effects of awe and thus explain the lack of effect on diversity measures.

### 4.1 Practical Implications

Organizations who offer diversity training to their employees on a voluntary basis may consider using happiness‐inducing methods when presenting it to their employees. Happiness‐inducing film clips in which the training package is advertised may be sufficient to increase the probability that employees sign up for the training. Presumably, more long‐lasting effects on diversity outcomes in the organization may be achieved by creating a joyful workplace with happy employees. However, whether happiness increases *diversity behaviour* in organizations or elsewhere needs to be examined in future research since behaviour may not follow automatically from attitudes, which was the focus of the current study.

### 4.2 Limitations and Future Directions

The current sample showed relatively low levels of SDO. This restriction of range may have limited the potential effect of the experimental manipulation. However, we found an effect of happiness, which suggests that the measure was sensitive enough to be impacted by the experimental manipulation.

Some degree of emotional overlap is expected in awe inductions, as awe is a more complex emotion with several interrelated emotions. According to Plutchik's (2001) three‐dimensional circumplex model, awe can be described as a mixture between surprise and fear. Nevertheless, one limitation of the current study is that it does manipulate negative awe vis‐à‐vis positive awe (cf. Gordon et al. 2017) to examine independent effects on diversity. Possibly, threat‐based (negative) awe could impede diversity. Gordon et al. (2017) have shown that threat‐based awe is imbued with fear, anxiety, and a sense of powerlessness. Since it has been established that fear and perceptions of threat fuel prejudice and discrimination (e.g., Kauff and Wagner 2012), it would make theoretical sense to assume that threat‐based awe could have a negative impact on diversity related outcomes. However, other studies (e.g., Piff et al. 2015) have found that negative awe, consisting of threatening stimuli, increases prosocial behaviour. Because prosociality is positively related to attitudes towards diversity (e.g., Beierlein et al. 2016), these findings may be potentially conflicting. Hence, there seems to be a need for future research to examine how threat‐based awe influences diversity behaviour. For example, in an experimental setup, positive and threat‐based awe could be induced, also using stimuli that are matched with respect to the broader context (e.g., space; see Gordon et al. 2017). The current study focused on positive awe since the typical awe experience does not contain threat and fear. However, threat‐based awe does not seem to be a rare phenomenon either, accounting for roughly one in five awe experiences (Gordon et al. 2017).

Finally, the observed effect sizes for our happiness manipulation are small. However, considering that we used brief film clips and measured relatively stable constructs (diversity attitudes and individual differences in SDO) that should not be easily manipulated, the effects are noteworthy. Whether more potent emotion inductions (including that of awe) that are repeated over time produce larger effects on diversity outcomes is an empirical question that should be addressed in future research.

## 5 Conclusions

Contrary to our hypotheses, people who are induced with awe do not seem to become more diversity‐oriented. The results suggest that happiness has greater potential to make people more diversity‐oriented. Future research should examine whether this happiness effect extends to actual diversity promoting behaviour in applied settings and to explore the potentially opposing effects of positive and threat‐based (negative) awe.

## Author Contributions

**Jens Agerström:** conceptualization, methodology, formal analysis, writing – original draft, writing – review and editing, investigation, project administration. **Magnus Carlsson:** conceptualization, methodology, formal analysis, data curation, writing – review and editing, investigation, funding acquisition. **Towe Nilsson:** methodology, formal analysis, data curation, software, writing – review and editing, conceptualization, investigation.

## Funding

This research was funded by the Swedish Research Council (grant nr. 2018‐03487).

## Ethics Statement

The authors have nothing to report. No ethical review board was consulted, since the Swedish Act concerning the Ethical Review of Research Involving Humans (2003:460) states that the types of research that shall be submitted to ethical review is research dealing with sensitive personal data as defined by GDPR article 9.1, research dealing with personal data regarding criminal offences, research involving a physical operation on the participant or a deceased person, research using a method intended to physically or psychologically influence the participant or where there is an obvious risk of physical or psychological injury, research involving biological traces from living or deceased people. As the current study does not belong to any of these categories, it is exempt from ethical review.

## Consent

Before participating in the study, all participants provided written informed consent, in accordance with the Declaration of Helsinki.

## Conflicts of Interest

The authors declare no conflicts of interest.

## Data Availability

The data that support the findings of this study are openly available in Open Science Framework at https://osf.io/uje7f/.

## Endnotes

^1^

https://osf.io/uje7f/.

## Appendix A See Table A1

**TABLE A1:** Main results with controls.

	(1)	(2)	(3)
	Attitudes towards diversity	Diversity fatigue	SDO
Awe	−0.002 (0.028)	−0.064 (0.049)	−0.053 (0.046)
Happiness	0.077*** (0.028)	−0.100** (0.049)	−0.144*** (0.046)
Female	0.150*** (0.023)	−0.488*** (0.040)	−0.417*** (0.038)
Age	−0.001 (0.005)	0.004 (0.009)	−0.025*** (0.009)
Age2	−0.000 (0.000)	0.000 (0.000)	0.000*** (0.000)
Constant	4.711*** (0.112)	2.008*** (0.193)	3.146*** (0.182)
*N*	3196	3196	3196
*R* ^2^	0.046	0.069	0.043

*Note:* This regression mirrors those in Table 2, but includes controls for gender, age, and age squared. See also the notes below Table 2. Robust standard errors in parentheses.

*** *p* < 0.01.

** *p* < 0.05.

* *p* < 0.1.
